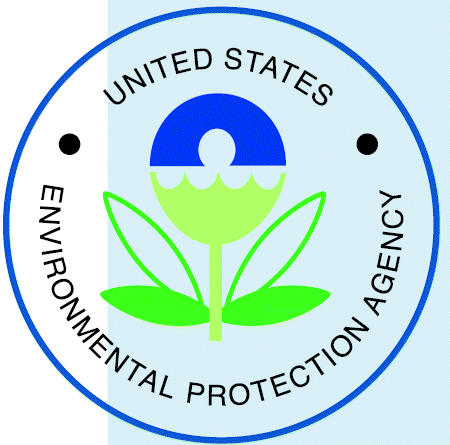# EHPnet: Environmental Technology Opportunities Portal

**Published:** 2004-09

**Authors:** Erin E. Dooley

In 2003, Congress mandated that the U.S. Environmental Protection Agency (EPA) set up a centralized office for facilitating public–private partnerships established to commercialize cost-effective environment-related technologies. As part of this effort, the EPA has created the Environmental Technology Opportunities Portal (ETOP), located at **http://www.epa.gov/etop/**, where technology developers can access the numerous programs—financial and otherwise—that the EPA offers them. The site is designed to help developers understand EPA programs on offer so that they can better take advantage of the money and other resources available through these programs.

The site has three primary sections. The largest is the For Technology Developers section. From this section, visitors can go to subsections to learn more about getting financial support, finding ways to demonstrate and verify their technologies, marketing their products, disseminating information, building partnerships, and advocating for their innovations.

The Financial Support subsection of this page includes information not just about EPA sources, but also about monies available from other federal agencies and the private sector. The Demonstration/Verification subsection has links to various programs designed specifically for field-testing and otherwise demonstrating new technologies in certain areas, such as the Superfund Innovative Technology Evaluation Program.

The Marketing subsection provides links to the VENDINFO database of pollution prevention equipment, products, and services, as well as to marketing/labeling programs such as Energy Star. Finally, the Information, Partnership, and Advocacy Programs subsection includes links to resources such as the EPA’s Technology Innovation Program, an information and advocacy group that promotes the use of new technologies in remediation of a variety of polluted sites. This program works with other federal agencies, states, engineering firms, responsible parties, investors, and developers to provide technology and market information and to facilitate the implementation of these innovations.

Back at the homepage, the Technology Users section of the ETOP site connects those searching for environmental technologies to appropriate solutions, sorted by type: air, water, solid and hazardous waste, and pollution prevention. Included is information on EPA research and development activities. This section also provides the Thesaurus of Environmental Technology Terms, a compendium of relevant terminology, technologies, programs, and offices.

The ETOP site offers a number of features on its homepage to help ensure that visitors can easily get the information they need. The Where You Live link leads to an interactive map that allows visitors to pull up information by EPA region or state (the map currently contains information just for Region 1). Visitors can also subscribe to two mailing lists: the ETOP mailing list provides information about funding opportunities as they are announced and updates to the ETOP site, while the EnvirotechNews mailing list features a calendar of upcoming events, information on federal funding opportunities, and items on enforcement actions. Finally, ETOP News pulls together news items of interest to the environmental technology developer community, such as the recent awarding of $900,000 to four companies to develop environmentally relevant nanotechnologies.

## Figures and Tables

**Figure:**